# The safety and efficacy of the tetanus vaccine intramuscularly versus subcutaneously in anticoagulated patients: a randomized clinical trial

**DOI:** 10.1186/1471-2296-15-147

**Published:** 2014-08-28

**Authors:** Fernando I Lago-Deibe, Maria-Victoria Martín-Miguel, Carmen Velicia-Peñas, Isabel Rey Gómez-Serranillos, Manuela Fontanillo-Fontanillo

**Affiliations:** Sárdoma Primary Care Health Centre, EOXI Vigo, Galician Health Service, Red Española de Investigación en Atención Primaria (Spanish Primary Care Research Network, REDIAPP), (Baixada a Laxe 76), Vigo, (36204) Spain; Matamá Primary Care Health Centre, Vigo Primary Care Region, EOXI Vigo, Galician Health Service, (Babio s/n), Vigo, (36312) Spain; Quality Unit, EOXI Vigo, Galician Health Service, (Rosalía de Castro 21-23), Vigo, (36201) Spain; Department of Statistics, Research Support Unit, EOXI Vigo, Spain

**Keywords:** Anticoagulants, Tetanus toxoid, Safety, Vaccines, Primary Health Care (Mesh Terms)

## Abstract

**Background:**

In patients treated with oral anticoagulants, subcutaneous injections of anti-tetanus vaccine are usually recommended to reduce the risk of bleeding, although the effectiveness of the vaccine has only been proven for intramuscular injection. The objective of this study was to compare the safety and efficacy of intramuscular and subcutaneous injections of tetanus-diphtheria vaccine in patients treated with oral anticoagulants.

**Methods/design:**

We present a prospective, double blinded, clinical trial comparing two groups of patients with oral anticoagulants: one group was administered tetanus-diphtheria vaccine by intramuscular injection, while the other was administered the same vaccine by subcutaneous injection. Allocation to each group was randomized and the duration of the study was six years.

Study population: all patients treated with oral anticoagulants, who had been administered with at least one dose of vaccine, at 15 Health Centres in Vigo (Spain), and who agreed to participate in the study. The sample size was 115 patients in each group. The main variables for the safety analysis were the measurement of the brachial diameter, the appearance of basic injuries at the vaccine administration site, the appearance of pain and systemic reactions. The variable used for the efficacy analysis was a significant increase in the titres of anti-tetanus toxoid antibodies.

An Intention-to-treat analysis will be performed. Details will be classified according to the administration route, while within each group a 3-tiered stratification will be defined by the administered number of doses. As a measure of association, relative risk will be estimated; the reduction of relative risk will also measured. For safety and to control the confounder effect, a logistic regression analysis will be carried out. As a measure of impact the reduction of absolute risk in relation to the total number of patients to be treated and the Number Needed to Treat will be estimated.

CONSORT 2010 guidelines were applied for reporting parallel group randomised trials.

**Discussion:**

The most significant difficulties on the project are related to the large number of participating centres, required to obtain a viable study population sample size, and the coordination given the scattering of the centres and researchers.

**Trial registration:**

ISRCTN69942081.

## Background

Despite its low incidence, tetanus is a major public health problem with a high fatality rate (40-50%) [1]. The annual register of cases has slowly been descending over the last 10 years, with an average of 25 cases/year in Spain (incidence of 0.06 cases per 100,000 population) [1]. In Galicia (Spain) during the last 5 years, on average, there have been 3 cases reported per year [2].

Tetanus is a disease that can be fully controlled, as it can be prevented by vaccination. However, it cannot be eradicated, because *Clostridium tetani* is a widely distributed microorganism in the environment. Immunization is highly effective [1], providing long-term protection and is recommended for the whole population in general, even though booster doses are required to maintain immunity, after the first vaccination [3].

Most cases of tetanus occur in previously unvaccinated adults, especially among those over 60 years of age [4]. Seroprevalence studies demonstrate that immunity from tetanus is higher than 95% in Spanish cohorts born after 1982, decreasing progressively in cohorts born prior to this date; for example, in the 1957–1966 cohort immunity is only around 55% [1]. Studies of the elderly have reported seroprevalence of 7.7% in those over 70 years of age [5]. In Spain, the fact that most adults of over 50 years of age have not been vaccinated or have only been incompletely vaccinated is probably because the immunization calendar for the five-dose course of tetanus vaccine was not introduced until the early 1970s [6].

With any wound, the need to administer active immunization (tetanus toxoid) alone or jointly with passive immunization (tetanus immunoglobulin) depends on the type of wound, the probability of contamination with tetanus bacillus, and also on the knowledge of the patient’s vaccination history [1]. The current recommended treatment is the combined tetanus-diphtheria vaccine (presentation for adults) or intramuscular Td vaccine [1–3].

Most anticoagulated patients in primary care consultations are seen due to auricular fibrillation and, on average, they are over 56 years of age [7, 8]. Hence, vaccination coverage is probably low [9].

In anticoagulated patients, because of the hypothetical risk of bleeding after injection, use of the intramuscular route has been traditionally discouraged, with the subcutaneous route being recommended, even for vaccines that are routinely administered intramuscularly, as for tetanus [10–12]. Indeed, in the literature, a few cases of major bleeding complications have been published [13]. Yet, although there is no uniformity of results in studies comparing the effectiveness of the two routes [14, 15], vaccine efficacy studies have used the intramuscular route [1, 10, 14], and subcutaneous administration may be less effective compared to intramuscular administration. Moreover, for most vaccines, local adverse reactions are more frequent with subcutaneous administration than with the intramuscular route [14–16].

The safety of the intramuscular route for the hepatitis B [17] and influenza vaccines [18], which are also administered intramuscularly, has been demonstrated in patients with alterations in coagulation and, consequently, the 2006 CDC guidelines recommend intramuscular injection for the tetanus-diphtheria (Td) vaccine depending on medical criteria [10].

We have not found any study in the literature that assesses the safety and efficacy of intramuscular (IM) and subcutaneous (SC) administration for the Td vaccine in patients treated with oral anticoagulants.

The objective of this study was to compare the safety and efficacy of the intramuscular and subcutaneous routes of administration for tetanus-diphtheria (Td) vaccine in patients treated with oral anticoagulants, and to verify the hypothesis that IM administration is safer and has greater efficacy.

## Methods/design

**Figure 1:**
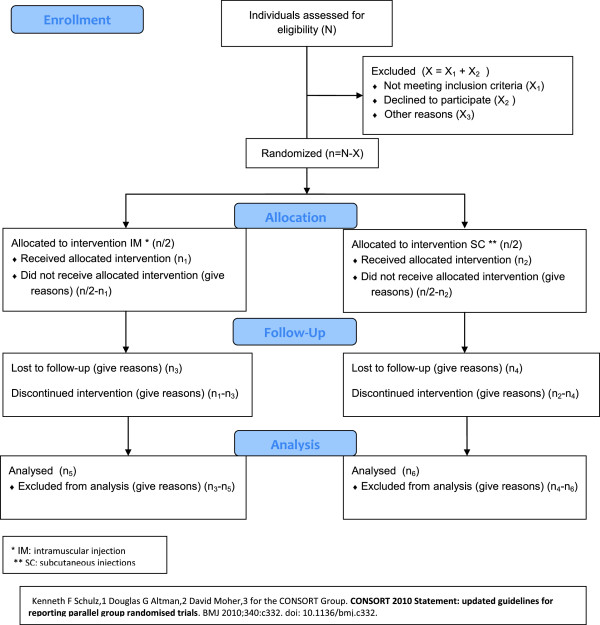
**Summary of trial design.**

We present a prospective, double-blind, phase IV clinical trial with two parallel groups and layered randomized assignment. Each one of these parallel groups was given doses of the corresponding tetanus vaccine, either intramuscularly (Group 1) or subcutaneously (Group 2), as shown in Figure 1. The layers were defined by the number of doses of the corresponding anti-tetanus injections (one, two or three), in each tier. This trial was only designed for the vaccination of the general anticoagulated population, and not for situations with wounds.

### Patients

The following inclusion criteria were applied to all patients who were undergoing monitored treatment with oral anticoagulants at 15 Health Centres within the Vigo Primary Care Area.

### Inclusion criteria

-  Patients treated with oral anticoagulants, for whom administering of at least one dose of anti-tetanus vaccine was indicated. This criterion was for those patients whose vaccination status was unknown, uncertain, or if they were clearly not vaccinated.
-   After being duly informed of the nature of the trial, patients were included if they gave written consent to be vaccinated and participate in the study.

### Exclusion criteria

-   Severe local reaction to previous doses with the whole circumference of the injected limb being affected.
-   Peripheral neurological disorders due to previous doses.
-   Severe anaphylactic reaction due to previous doses or any of the components.
-   Poor haematological control (International Normalized Ratio [INR] > 4) in the last 2 months.
-   Serious illness, terminal stages of diseases, immobilized, adversely affected by chronic pathology or immunosuppressive states.
-   Pregnant or breast-feeding women.

### Sample size

Assuming that for the IM route the percentage of local side effects is 30% [11, 15, 16] and expecting an increase of 18% [15] in local secondary effects for the alternative route (SC), using a bilateral approach, with a 95% confidence interval, a beta risk of 0.20 requires 115 patients in each group. Taking into account the possible 15% loss in information, the final sample size was 135 patients in each group. With this sample size, it was estimated that a 3 UI/ml mean difference in antibody levels could be detected.

### Interventions

Patients were recruited at the primary care centres by their family doctors. During the first visit, the doctor assessed the vaccination state of the patient, taking into account the vaccination records in the patient’s medical history, or by interviewing the patient if such information was not available. According to the number of doses received and the date of the last dose, the doctor was able to determine if the patient was properly vaccinated (and, if so, the patient was excluded from the study), or whether they needed to be given a booster dose, or to start or complete adult primo-vaccination. The guidelines used for applicable vaccination were those recommended by the Spanish Ministry of Health in 2008 [1, 3, 4]:

For complete primo-vaccination, and less than 10 years since the last dose: nothing (not included in the study).

- For complete primo-vaccination, and more than 10 years since the last dose: 1 booster dose.
- In cases of no previous vaccination: complete primo-vaccination with three doses separated by 1–2 months between the first two, and 6–12 months between the second and third, with subsequent booster doses every 10 years. If primo-vaccination had been started prior to the beginning of the study, the patient was administered doses according to the standard schedule.
- In cases of incomplete primo-vaccination:
  - One dose if the patient already had been administered two doses, and the last dose was more than a year earlier.
  - Two doses, separated by six months, if the patient had one dose administered more than a month earlier.

When vaccination status was unknown or doubtful, primo-vaccination was started.

After assessing if the patient fulfilled all inclusion criteria, and none of the exclusion criteria, they were invited to participate in the study and, if they agreed to sign informed consent, were included in one of the tiers, according to the corresponding (allocation ratio 1:1) vaccine dose (one, two or three). The patients were given an appointment to have an INR test and, if the score was less than 4, a blood sample was taken to determine anti-tetanus antibodies, and a dose of vaccine was immediately administered, so that the patient could proceed to the corresponding nursing consultancy. The nurse was responsible for requesting from the randomization centre, by telephone, the administration route to which the patient had been assigned*.* The patient was not informed of the administration route used*.* Patients requiring more than one dose received all doses via the same route, and were given appointments in relation to the interval and number of corresponding doses.

The doctor was blinded to the administration route and also to the follow-up appointments (1, 2, 14 and 30 days) after each dose of the vaccine to detect any side effects.

On the first visit, after administering the vaccine, and at 30 days after the last dose of anti-tetanus vaccine (ATV), the antibody titre was determined for all patients using enzymatic immunoanalysis.

The systematic physical examination at each visit included the following aspects:

-  General appearance.
-  Temperature
-  Arterial blood pressure.
-  Measurement of the brachial perimeter at the height of the deltoid on the first visit, and at the site of inoculation after vaccination.
-  Examination and palpation of the injection site looking for basic injuries.
-  Homolateral axillary palpation of the injection site.
-  Any examination required due to the emergence of a general and/or unexpected side effect.

### Laboratory analysis

-   Determination of INR through the capillary technique with a reflectometer.
-   Determination of antitoxoid tetanus antibodies by enzymatic immunoanalysis in a centralized Laboratory (Barcelona).

### Data collection

The first patient was included in January 2009, with an initial 24-month forecast for the period of inclusion, which subsequently had to be extended to 72 months, due to difficulties in reaching the predetermined sample size

All data were recorded on a case report form (CRF) designed for that purpose. A specific database was created to upload the data.

A training workshop was held for all researchers participating in the clinical trial regarding techniques, collection of data and measurement of study variables prior to the beginning of the study.

### Premature abandonment by a patient

The researcher determined the primary reason for premature abandonment by the patient and recorded this information on the CRF. Patients could be prematurely removed from the study for one of the following reasons: side effects in primo-vaccination; patient withdrew consent; loss of follow-up; administrative problems or death.

For patients who could not be contacted and missed the follow-up, the researcher documented the steps taken to contact the patient.

### Variables

The main analytical safety variables were:

1. Measurement of the brachial perimeter in centimetres.
2. Appearance of basic injuries (redness, swelling, heat, granulomas, haematoma) in the area of administration of the vaccine, axillary nodes, and the appearance of pain measured with the visual analogue pain scale.
3. The emergence of general symptoms (fever, malaise, headache, weakness, arthralgias).
4. The appearance of any serious adverse effect: one that was fatal or posed danger to patient’ life, ended in disabilities or required hospitalization.

For each route of administration, the main variable used in the efficacy analysis was the increase in anti-tetanus antibodies following vaccination.

### Statistical analysis

Descriptive, statistical analysis will be used to classify the data into two groups, according to the route of administration, while within each group a 3-tiered stratification will be defined by the administered number of doses. The quantitative data will be summarized by estimating the arithmetic mean and median as measures of central tendency, as well as the standard deviation as a measure of dispersion. For qualitative variables, proportions with 95% confidence intervals will be estimated.

After checking the conditions of application and assuming an alpha error of 0.05, the Chi-squared test will be used to determine the statistical differences between the qualitative variables in the two study groups. Prior checking of all the criteria for application will be carried out before the Student’s-t test (0.05 alpha) is used to examine the levels of significance in the differences in quantitative variables for independent data. If the conditions of application were not met, the non-parametric Mann–Whitney U test will be used. The Student’s t test for paired data will be employed for paired quantitative variables.

As an association measurement, relative risk will be estimated with a 95% confidence limit, according to the method of Mantel and Fleiss. In addition, as a further association measurement, relative risk reduction will be calculated with a 95% confidence interval. For the safety study and to control the effect of different confounding covariables, a logistic regression analysis will be simultaneously performed.

As a measurement of impact, the reduction of absolute risk (RAR) and the Number of patients Needed to be Treated (NNT) to prevent an event will be calculated with 95% confidence intervals.

An Intention-to-treat (ITT) analysis will be carried out, even when it was highly or totally improbable that crossing occurs within groups, because this intervention is predictably limited to a particular moment.

### Allocation and masking

The randomization unit is the individuals participating in the trial. A random allocation is made within a 3-tiered stratification based on the number of doses of vaccine required for successful immunization (1, 2 or 3 doses). Within each tier, simple randomization is performed by spreadsheet, in an attempt to control the confounder effect of the number of doses.

Mapping is performed by the Fundación Galicia Sur (EOXI Vigo) to which all researchers were given telephone access.

Information concerning the randomization process remained confidential until the end of the study. It is the responsibility of each researcher to ensure that there is a specific procedure that allowed for the opening of the code in case of emergency, with immediate communication to the randomization centre.

The route of administration is masked in the database to prevent the team performing the computer analysis from discovering the route that corresponded to each group.

Loss of masking only occurred in cases of emergency for the patient and at the conclusion of the study.

### Ethical aspects and confidentiality of data

In compliance with the Helsinki Declaration, it was the responsibility of the researcher to inform the patients of their participation in a clinical study, and to clarify that this participation was voluntary and did not imply any change in treatment or medical care received by participating patients, as compared to that received by patients who did not participate. The researcher then had to obtain written, informed consent from patients before they could be included in the study.

The highest levels of confidentiality and professional conduct were always maintained and the existing national legislation on data protection was respected. The patients’ identities were encoded in the study documents and only duly authorized personnel had access to identifiable personal data, when data verification procedures required that this information had to be inspected.

It was the responsibility of the researcher to inform the patients in a clear and concise way that their data would be incorporated into a computerised database, which would only be used for clinical research purposes, and that the patient could not be identified in that database. In the database, the patients were each assigned an internal code number by the randomization centre, which remained unknown to the researcher, so that their clinical data could not be associated to the identified or identifiable person.

Likewise the CRF data were treated confidentially. The name of the patient was hidden and could only be identified through a number corresponding to the researcher code and the patient code.

This project respected ethical principles for medical research involving human subjects, as set out in the World Medical Association Declaration of Helsinki, together with the clinical research rules of the Spanish Research Act Regulations, and was granted permission by the Clinical Research Ethics Committee with approval obtained on 07/06/2007 with number 2007/089 (N° EudraCT 2007-001073-29), which was subsequently modified and extended on 13/04/2009, 10/09/2009 and 13/12/2010 by successive expansion in the number of participating health centres (from the initial 6 to 15), to improve recruitment of patients.

During the development of the study, there was regular monitoring that the information contained in each patient’s CRF was complete and regularly updated, and that the required good clinical practice was being maintained in adherence with the Protocol and the follow-up procedures.

The original documents of each participating patient in the study, consisting of annotations made during each visit and the signed original of informed consent forms, were maintained by the researchers.

## Discussion

This present clinical trial was designed to demonstrate that the administration of tetanus vaccine via the intramuscular route is safe and has greater efficacy than the subcutaneous route in anticoagulated patients, which would make it possible to routinely recommend this route in these patients. This is relevant because the prevalence of patients treated with oral anticoagulants is increasing, even at younger ages, and anti-tetanus vaccine, the only one with universal indication, is still the only measure with proven efficacy for preventing new cases of tetanus.

There are inherent difficulties in conducting clinical studies, with a variety of possible eventualities that affect researchers, patients and the actual structure and organization of the centres. To these handicaps, must be added the following particular characteristics of primary care: the extra effort required for coordination given the scattering of the centres and researchers; the accessibility of care at this level, at least in Spain, which causes great pressure on health and welfare services with little control over the distribution of daily work, with a resulting conflict regarding the space and time available for research activities.

In addition, there are problems relating to the study itself. For example, as the number of anticoagulated patients with vaccination criteria is low, to obtain a viable study population sample size required the participation of a large number of centres, which hindered the monitoring and coordination of the project. Finally, the large number of participating centres increased the need for special care in keeping doctors and patients blind to the route of vaccination.

## Abbreviations

Td: Tetanus-diphtheria (vaccine)
IM: Intramuscular
SC: Subcutaneous
INR: International normalized ratio
CRF: Case report form
NNT: Number (of patients) Needed to be Treat to prevent an event
ITT: Intention-to-treat (analysis)
RAR: Reduction of absolute risk.
